# “We are part of a family”. Benefits and limitations of community ART groups (CAGs) in Thyolo, Malawi: a qualitative study

**DOI:** 10.7448/IAS.20.1.21374

**Published:** 2017-03-28

**Authors:** Umberto Pellecchia, Saar Baert, Spencer Nundwe, Andy Bwanali, Bote Zamadenga, Carol A. Metcalf, Helen Bygrave, Sarah Daho, Liesbet Ohler, Brown Chibwandira, Kennedy Kanyimbo

**Affiliations:** ^a^ HIV project, Médecins Sans Frontières, Thyolo, Malawi; ^b^ Médecins Sans Frontières, Southern Africa Medical Unit, Cape Town, South Africa; ^c^ Country office, Médecins Sans Frontières, Blantyre, Malawi; ^d^ Ministry of Health, District Health Office, Thyolo, Malawi

**Keywords:** HIV, ART delivery, differentiated care, task-shifting, service delivery, Malawi

## Abstract

**Introduction**: In 2012 Community ART Groups (CAGs), a community-based model of antiretroviral therapy (ART) delivery were piloted in Thyolo District, Malawi as a way to overcome patient barriers to accessing treatment, and to decrease healthcare workers’ workload. CAGs are self-formed groups of patients on ART taking turns to collect ART refills for all group members from the health facility. We conducted a qualitative study to assess the benefits and challenges of CAGs from patients’ and healthcare workers’ (HCWs) perspectives.

**Methods**: Data were collected by means of 15 focus group discussions, 15 individual in-depth interviews, and participant observation in 2 health centres. The 94 study participants included CAG members, ART patients eligible for CAGs who remained in conventional care, former CAG members who returned to conventional care and HCWs responsible for providing HIV care. Patient participants were purposively selected from ART registers, taking into account age and gender. Narratives were audio-recorded, transcribed, and translated from Chichewa to English. Data were analyzed through a thematic analysis.

**Results**: Patients and HCWs spoke favourably about the practical benefits of CAGs. Patient benefits included a reduced frequency of clinic visits, resulting in reduced transportation costs and time savings. HCW benefits included a reduced workload. Additionally peer support was perceived as an added value of the groups allowing not only sharing of the logistical constraints of drugs refills, but also enhanced emotional support. Identified barriers to joining a CAG included a lack of information on CAGs, unwillingness to disclose one’s HIV status, change of residence and conflicts among CAG members. Participants reported that HIV-related stigma persists and CAGs were seen as an effective strategy to reduce exposure to discriminatory labelling by community members.

**Conclusions**: In this setting, patients and HCWs perceived CAGs to be an acceptable model of ART delivery. Despite addressing important practical barriers to accessing ART, and providing peer support, CAGs were not well known by patients and had a limited impact on reducing HIV-related stigma. The CAG model of ART delivery should be considered in similar settings. Further measures need to be devised and implemented to address HIV-related stigma.

## Introduction

In 2014, the United Nations Programme on HIV/AIDS (UNAIDS) set ambitious targets, for reducing the global impact of HIV, aiming at 90% of people infected with HIV being aware of their status; 90% of those diagnosed as having HIV infection being retained in care; and 90% of those in care being virologically suppressed, by 2020 [1]. Retention in care has proven to be challenging, with overall low retention figures in resource-limited settings [2]. The shift to “Test and Treat” recommended by WHO in its 2015 guidelines [3] will further increase the number of people starting ART and needing to be retained in care.

Healthcare systems are confronting the challenges of initiating growing numbers of patients on ART and retaining them in care. A shift towards differentiated models of care for people living with HIV is becoming increasingly important [4], whereby community-based ART delivery strategies for stable patients on ART will be key to meeting the 90–90–90 targets [5,6].

The Community ART Group (CAG) model is one such example of a differentiated model of care, which delinks clinical consultations and ART refills among stable patients on ART. CAGs are self-formed groups of people living with HIV. CAG members take turns to collect ART refills for all group members from the health facility, on a rotational basis, while continuing to have clinical consultations and blood taken for viral load testing according to local guidelines [5]. The CAG model was developed in rural areas of Tete Province, Mozambique, where it had a favourable impact on retention in care [7,8] and a high level of acceptance from patients and healthcare workers [9].

Following the successful implementation of the CAG model in Tete, the Thyolo District Health Office (DHO) in Malawi and Médecins Sans Frontières (MSF) adapted the model and introduced CAGs in Thyolo District, Malawi in 2012. The model was implemented in order to address challenges such as long waiting times in clinics, long distances to the health facilities for patients, staffing shortages and heavy healthcare worker workloads. Outcomes of the model in terms of clinic attendance and retention in care have been described elsewhere [10]. Here, we report the findings of a qualitative study to assess the perceived benefits and limitations of CAGS from a patient and a healthcare worker (HCW) perspective.

## Methods

### Study setting

Thyolo, a rural district in Southern Malawi, has a high burden of HIV, with an HIV prevalence of 14.5% (National Statistical Office, 2010). Since implementation of the ART programme in 2003, 69,289 people have been started on ART, with 41,483 in care in June 2015. HIV care is provided at 30 health facilities, and ART refills are provided 1 to 3-monthly by nurses or health surveillance assistants (HSAs), a cadre of community health workers. CAGs were piloted in four facilities, starting in 2012, and rolledout to 15 out of the 30 health facilities in the district by June 2015. By June 2015, 825 CAGs were operating, providing care for 4,933 patients [11].

The study was performed in the two of the CAG pilot sites: Mikolongwe Health Centre with 27% of a cohort of 2,120 HIV-positive patients on ART in CAGs, and Khonjeni Health Centre with 23% of 3,038 HIV-positive patients on ART in the second quarter of 2015.

### CAG intervention

The CAG model of differentiated ART delivery was implemented in Thyolo district through the Thyolo DHO with support from MSF. DHO staff, such as the district ART coordinator, were assisted by MSF CAG officers in training and supervision of healthcare workers, and monitoring and evaluation of the CAG intervention. The National Association of People living with HIV/AIDS in Malawi (NAPHAM) who facilitate HIV support groups in Thyolo district, was involved from the outset to support the promotion and formation of CAGs.

CAGs are promoted both at the facility and in the community through health talks given by Health Surveillance Assistants (HSAs), Malawi’s community health worker cadre, and by NAPHAM support group leaders. Stable patients on ART are invited to form a group of their choice with other patients on ART from their community. Each CAG elects a focal person to lead the group. From all the CAG focal persons from a defined catchment area a CAG leader is chosen. This person assists the CAG focal persons and liaises with the HSA of the area should further support be required for an individual group.

After screening for CAG eligibility by the nurse or clinician, CAG members meet monthly at the home of a member or other chosen community venue to exchange on members’ adherence and other issues and fill in the CAG community card. A CAG representative is chosen who then reports to the health facility and collects ART for the rest of the group. While at the clinic the CAG representative receives a clinical consultation and blood tests as required by the nurse or clinician. The representative then returns to the community to distribute the ART to the group members.

A more detailed description of the Thyolo CAG model can be accessed at http://samumsf.org/blog/portfolio-item/lessons-learnt-in-thyolo-malawi-from-implementing-cags/

### Study design

The study was carried out between May and August 2015. Patient and HCW perspectives of CAGs were assessed by means of 15 focus group discussions (FGDs), 15 individual in-depth interviews (IDIs), and 2 days of participant observation in the two study health facilities. Different qualitative data-collection methods were used to gain a broader understanding and allow cross-validation of data.

### Study participants

A purposive sampling technique was used, applying principles of maximum variation and gradual selection [12]. For patients gender, age and type of care received were taken into account, while for healthcare workers their role in the health facility guided the selection of study participants. Participants included 52 CAG members, 8 ex-CAG members who had returned to conventional care, and 9 patients on ART eligible for a CAG who had elected to remain in conventional care. Study participants were identified through CAG registers and during participant observation in the health facilities. The sample of HCWs included 5 Medical Assistants, 2 Nurses and 18 Health Surveillance Assistants (HSAs).

Potential participants were identified and approached in the health facilities. The rationale and modalities of the study were explained to them individually and a date for a FGDs or IDIs was scheduled with those who consented to participate.

### Data collection

The study was led by an anthropologist external to the study district, and was supported in the data collection, transcription, and translation by four native Chichewa speaker research assistants. FGDs and IDIs were conducted in English or Chichewa, depending on the study participants’ preference.

Interview guides were developed for the FGDs and IDIs, based on the study questions. An iterative process was applied whereby information gathered during the study led to the formulation of new questions, until saturation of new information was reached.

All FGDs and IDIs were audio-recorded and transcribed. Translation into English was done when needed, and a sample of fifteen per cent of the English transcripts of FGDs and IDIs conducted in Chichewa was back-translated and reviewed for consistency by a third neutral professional translator.

### Data analysis

A thematic analysis of the data was done by the principal investigator and led to a manually developed coding framework grouped into three broad interrelated categories: medical aspects, functioning of the model of care, and social aspects; which were each divided into sub-categories.

### Ethical considerations

Ethical approvals were granted by the MSF Ethics Review Board (Protocol 1307) and the Malawi National Health Sciences Research Committee (NHSRC #1150) in 2015. Written consent, in English or Chichewa, was obtained from all study participants prior to data collection. Participants were reimbursed for transport costs incurred as a result of participation in the research.

## Results

### Benefits of joining a CAG: practical benefits and peer support

Practical benefits seemed to be the main driver for patients on ART to join a CAG. Participants in CAGs identified the main reasons for joining a CAG as the reduction in time spent getting to and waiting at the clinic for ART refills, reduced transport costs and opportunity costs linked to time lost regarding work.

These practical benefits of CAGS were also identified by participants eligible for CAGs who were in conventional care, and who had not been aware of the option to receive ART through the CAGs prior to this study.

*The groups are very good. If I go to the hospital this month, then I will go again after five months. I can spend this time concentrated on my work and businesses, in the garden, watering the vegetables*. (Female CAG member in FGD)

HCWs welcomed the implementation of CAGs because of the impact that they had on reducing congestion in health facilities and reducing their workload.

Some medical assistants and nurses reported that CAGs had enabled them to improve their relationship with patients, leaving them more time to consult patients in need. However, none of the patient participants mentioned an improvement in their relationship with HCWs due to CAGs, nor saw this as a reason to join CAGs, although some patients observed that CAGs had reduced pressure on HCWs.

*At first, I had lots of patients and, instead of having time for them, I was busy looking at how many patients were left outside. This time around, instead, because of this CAG the congestion is reduced per day and we are able to see a countable number of patients for which we have enough time*. (Male medical assistant in FGD)

CAG members reported that they valued the peer support that they received through CAGs. This peer support had enabled them to overcome barriers to retention in care, by sharing the task of collecting ART refills, and had also been a source of emotional support by sharing the burden of being an HIV positive person. However, most of the CAG members who reported experiencing high levels of peer support belonged to groups formed from support groups and belonging to the National Association for People living with HIV and AIDS in Malawi (NAPHAM), and had known the other members of the group prior to forming a CAG.

*[The] group is good, it is like a family, doing things together like a family, understanding each other*. (Female CAG member in FGD)

Adherence support was often provided in CAG meetings. This included discussing physical ailments and members’ ability to accomplish daily tasks. Health was socially conceived and participants generally did not refer to medical concepts, such as viral load to evaluate one’s health.

*Yes! I feel that the drugs are having power on me. You can see me, I am in good health, I have no worries and those who used to laugh at me now stopped […] I have built this beautiful house and even if I die now I am sure my kids will have a good house!* (Male CAG member in IDI)

Some participants reported that there were economic benefits to being in a CAG. Taking the opportunity of having formed a group, certain CAGs spontaneously started income-generating activities by investing as a group in small livestock. Although income-generating activities are not formally part of the CAG’s functions, it was one of the aspects of being in a CAG highly valued by study participants, while at the same time being a potential source of conflict within the group.

### Barriers to being in a CAG

While acceptability of CAGs was high among CAG members, patients in conventional care and healthcare workers who were not directly involved in CAGs, generally had little knowledge or awareness of CAGs and CAG functioning. Some participants who were in conventional care only learnt about CAGs through the study, and were keen to join a CAG once they learned about them. This was mirrored by observations at the health facilities showing a lack of promotion of the model.

Some participants who were aware of CAGs, but who had remained in conventional care identified additional barriers to joining a CAG. The act of joining a group implies that individuals have already voluntarily disclosed their HIV status to others, or are willing to do so. Some participants who had chosen to remain in conventional care preferred not to disclose their status to others.

*There are some people who don’t want to come and join groups because they don’t want to disclose their status […] they feel shy and we meet them and explain that their problem can easily be resolved in the group*. (Female CAG member in FGD)

Some participants with weak social networks, such as those who had come from outside the district for work, or who were not part of an existing support group, reported that it could be difficult to establish a CAG.

Former CAG members who had left a CAG reported that they had done so because of group and gender dynamics, or mobility. While many CAG members referred to their group as being like a family, some CAGs had collapsed due to interpersonal conflicts, such as tensions among friends or in couples. These participants reported that they had subsequently found it hard to re-form a group, and preferred to return to conventional care. They did suggest the HSA or local chief could potentially assist CAGs to resolve such conflicts. Gender dynamics were also reported to have led to the collapse of some groups, as the equal role men and women have in a CAG was at odds with the traditional patriarchal role that men have in the household. One of the most important reasons given for leaving a CAG was moving away from the area in search for work.

### Functioning of CAGs: do CAGs work as intended?

Participants who were CAG members, and healthcare workers showed a good comprehension of the functioning of the groups with its rotation system, reporting tools and system of referrals back to the health facility.

Although it was not policy, participant observations showed that CAG members received a preferential treatment. They were prioritized by health workers when dispensing ART refills, making their waiting time at the clinic shorter than for patients who remained in conventional care.

Pre-set communication channels exist for CAG members to flag problems within the group, such as through the chosen CAG leader for a particular region. Participants reported that the person who is requested by the group to problem solve is often someone considered socially more respected such as the Health Surveillance Assistant (HSAs) or the MSF CAG officer. Participants requested a broader role from these lay cadres, asking the HSAs to be the link between the CAG and the health facility, and to enhance group dynamics and solve problems in case they arise.

HSAs were observed to play an important role in assisting CAG functioning and in the drug distribution at the health facilities. HSAs did, however, report a lack of clarity of their role in CAGs, due to poorly defined supervision lines regarding this activity, and the wide range of tasks assigned to HSAs, apparently without consideration being given to the feasibility of HSAs meeting the demands.

### Less stigmatization or less discrimination?

Participants reported that they frequently experienced discrimination within their communities, but that this had decreased over time as more people experienced HIV within their households. They cited labels used by community members to identify PLHIV, reflecting the negative attitude towards people who are no longer able to take care of themselves or who are incapable to work due to HIV. Participants in turn performed self-protective acts to hide their status, as a way to normalize their social status. Participants in CAGs explained how CAGs became an effective strategy to reduce exposure to discriminatory acts. Less clinic visits reduced the possibility of CAG members being identified by community members as having HIV infection. According to the model as conceived, CAG members have a group meeting for drug distribution, once the CAG representative returns from the clinic. However, the study found that many CAG representatives delivered the drugs house-by-house, in order to avoid members’ HIV status becoming known within the community.

*We quarrelled with other patients who were telling us ndife akufa kale, meaning ‘you are already dead’. This was a big problem […] CAG has reduced it very much, because they don’t see us so often anymore*. (Female CAG member in FGD)

Some CAG members had developed a social consciousness about HIV, which had enabled them to speak out openly about HIV, and to encourage others to disclose their HIV status. Most of those who felt comfortable about speaking openly about HIV were NAPHAM members.

## Discussion

The CAG model has been shown to be an acceptable model of ART delivery, for people on ART and healthcare workers providing ART services. Through the development of a patient-centred model of ART refill, it was possible to address the main barriers to access ART for patients, with the majority valuing the practical benefits of CAGs in reducing the frequency of clinic visits and the associated transportation costs. Additionally, peer support and in some cases income-generating activities were seen as an added value of CAGs. These findings are in line with those of similar studies performed in Mozambique and Haiti, showing cost and time savings and mutual peer support through CAGs [9,13]. Similarly, other differentiated models of ART delivery have shown to be beneficial for patients [5].

CAGs were also able to reduce the burden on healthcare workers due to fewer consultations. A quantitative evaluation of the Thyolo CAGs confirmed that the frequency of clinic visits decreased after patients join a CAG [10].

The practical benefits of sharing the logistical constraints for ART collection and facilitating patients retention in care is mirrored in a quantitative analysis of this model in Malawi [10] and Mozambique [14], and was also observed in a study of a similar model of ART refill distribution, conducted in South Africa [15].

Several CAG members who participated in the Thyolo study claimed that their adherence to ART had improved because of the peer support that they had received in the CAG. This needs to be confirmed by quantitative research comparing viral load among CAG members and patients in conventional care. The adherence club model in South Africa does suggest this may be the case [15].

The findings of this study suggest a number of ways in which the CAG model could be further adapted in order to ensure that it reaches its full potential. Several of the participants in conventional care, interviewed in this study, were not aware of the existence of CAGs, but showed an interest in joining a CAG once they learned that joining a CAG was an option open to them. Similarly in Kenya a lack of promotion of differentiated models for ART delivery was a barrier to a higher uptake by patients [16]. Better promotion of such models at the health facility level, and more broadly through HIV support networks, is needed to ensure patients demand services which are better adapted to their needs. At the same time healthcare workers need to be held accountable for the rollout of such models, once they become national policy.

This study found that CAG members were being given preferential treatment at the health facility. Similar observations were made with an evaluation of CAGs in Tete, Mozambique [9]. While such practices are sometimes introduced in order to provide an incentive for patients to join CAGs, such strategies need to be halted in light of providing equal access to services for all patients on ART.

This study found that adherence support formed an important part of the exchanges among peers in CAG meetings, which are often triggered by the observation of lack of physical strength, reduced capacity to perform daily tasks and loss of their social identity. These triggers have also been identified by others as an important barrier to adherence and source of stigmatization in Sub-Saharan Africa [17]. The lack of patient awareness of indicators of adherence, such as viral load or CD4, shows that a greater investment needs to be made in patient education when patients start treatment, to ensure that patients on ART have the tools to act early when experiencing adherence problems and avoid potential physical weakness and its social consequences.

Patients request a stronger involvement of lay cadres, such as the Malawi HSAs. Such cadres have been identified as a critical enabler for the scale-up of differentiated models of care [4–6]. As lay cadres are well recognized by the community and do community outreach activities, their role in differentiated models of ART distribution urgently needs to be formalized in Sub-Saharan Africa, where these lay cadres often lack recognition and sustained financing [18].

CAGs require members to be resident within the catchment area of a specific facility that provides ART refills. Some of the patients receiving ART from the two facilities in our study were mobile as they depended on seasonal work in the local tea plantations. An adapted ART refill strategy which is nationally endorsed is thus needed to ensure access to ART for patients who migrate in and out of the area.

CAGs have shown to be a successful strategy to improve ART delivery, but also have the potential to play a bigger role in establishing comprehensive care and support for people with HIV infection, and to address structural barriers to health such as poverty and stigma. Some CAGs in our study, had established income-generating activities; some participants expressed a desire to play a more important role in their communities; and CAGs formed from existing local support groups were easier to establish and had been vocal in confronting HIV-related stigma. To ensure true patient empowerment, a bigger investment in community models of care is needed, supported by strong networks of HIV-infected people. At a time of withdrawal of funding for civil society and community-based organizations, an important opportunity may be missed.

Limitations to this study include the following: Participant observations as a way to understand perceptions, practices and attitudes of different actors were done at the health facilities only. Carrying out participant observation in other settings beyond the facility may have provided a more in-depth understanding of the daily realities of patients and healthcare workers. Second, this study was performed in a setting where the CAG model, as one of several possible models for differentiated ART delivery, had already been implemented and promoted in the study area. It would be useful to conduct formative qualitative research to determine the preferences of patients and healthcare workers regarding differentiated models of ART delivery before implementation, in order to ensure contextual adaptation of any ART delivery model.

## Conclusions

CAGs were regarded as a highly acceptable model for ART delivery among patients and healthcare workers. CAGs addressed patients’ practical barriers to accessing ART and improved peer support, a factor patients considered fundamental to their wellbeing. However CAGs had a limited impact on reducing HIV-related stigma. To maximize the impact of this model adequate planning and monitoring of its implementation is required from the facility. Further expansion of this and other differentiated models of ART delivery should be considered, to achieve the ambitious 90-90-90 targets.
